# Improved Mask R-CNN for Aircraft Detection in Remote Sensing Images

**DOI:** 10.3390/s21082618

**Published:** 2021-04-08

**Authors:** Qifan Wu, Daqiang Feng, Changqing Cao, Xiaodong Zeng, Zhejun Feng, Jin Wu, Ziqiang Huang

**Affiliations:** 1 School of Physics and Optoelectronic Engineering, Xidian University, 2 South Taibai Road, Xi’an 710071, China; qfwu_1@stu.xidian.edu.cn (Q.W.); xdzeng@xidian.edu.cn (X.Z.); zhjfeng@mail.xidian.edu.cn (Z.F.); jinw9824@stu.xidian.edu.cn (J.W.); zqhuang_1@stu.xidian.edu.cn (Z.H.); 2 Shandong Institute of Space Electronic Technology, Yantai 264670, China; fengdq_0109@126.com

**Keywords:** Mask R-CNN, self-calibration, DOTA dataset, aircraft, remote sensing image

## Abstract

In recent years, remote sensing images has become one of the most popular directions in image processing. A small feature gap exists between satellite and natural images. Therefore, deep learning algorithms could be applied to recognize remote sensing images. We propose an improved Mask R-CNN model, called SCMask R-CNN, to enhance the detection effect in the high-resolution remote sensing images which contain the dense targets and complex background. Our model can perform object recognition and segmentation in parallel. This model uses a modified SC-conv based on the ResNet101 backbone network to obtain more discriminative feature information and adds a set of dilated convolutions with a specific size to improve the instance segmentation effect. We construct WFA-1400 based on the DOTA dataset because of the shortage of remote sensing mask datasets. We compare the improved algorithm with other state-of-the-art algorithms. The object detection AP_50_ and AP increased by 1–2% and 1%, respectively, objectively proving the effectiveness and the feasibility of the improved model.

## 1. Introduction

With the development of the remote sensing technology, researchers can obtain higher-resolution remote sensing images, showing broad application prospects in civil and military applications [1,2,3,4]. However, object detection and segmentation in high-resolution remote sensing images have always been a puzzle because of the large field-of-view of remote sensing images and dense targets.

The complex background of remote sensing images and dense targets bring great challenges to object recognition and segmentation. Traditional object detection algorithms have disadvantages, such as weak generalization ability and poor rotation invariance. The rapid development of deep learning (DL) provides a superior solution to this problem. A convolution neural network (CNN) has excellent effects in object detection, image generation, semantic segmentation, and super-resolution image reconstruction. As one of the most important directions of DL, object detection principally solves basic vision problems, such as classification and location of various targets in images. In the past 10 years, the system of CNN is continuously improved and enriched because scholars proposed many classical models and structures, such as region-based CNN(R-CNN), Fast R-CNN, SPP, FPN, FCN, and YOLO [5,6,7,8,9,10,11,12,13]. Some of these methods have been used by later scholars. In 2015, Girshick proposed Fast R-CNN. In the same year, Ren et al. proposed Faster R-CNN [12], which proposes a region generation network (RPN) to replace the previous selective search algorithm, greatly reducing the cost of candidate region generation. In 2016, Liu et al. proposed SSD, which achieves a good real-time performance without losing more accuracy [14]. The detection effect of the CNN model applied to a remote sensing image aircraft target has been found to be better than traditional methods [15,16,17,18]. Other types of remote sensing object detection techniques based on the CNN model have also achieved good results [19,20,21]. Yuan et al. [18] and Wu et al. [22] achieved a rapid detection of aircraft targets based on YOLOv3 and YOLOv3-tiny, respectively. Wang et al. [14] proposed a method of replacing the original VGG-16 with densely connected networks as a backbone network and built a feature pyramid between densely connected modules to replace the original multi-scale feature maps based on the SSD detection framework. These also confirm the superiority of deep learning compared to traditional machine learning methods.

In 2017, He et al. proposed Mask R-CNN [23]. The model is based on Faster R-CNN. In addition, the depth of networks has a great influence on the final effect for a deep learning network. The ResNet101 is a deep backbone network which can represent more complex functions and learn features from different network levels from edges (lower layers) to very complex features (deeper layers). Mask R-CNN chooses ResNet101 as its backbone network. For the original classification and bounding-box regression branches, a mask branch, which can simultaneously achieve object detection and instance segmentation, is added. Zhang et al. [24] proposed a ship identification method based on scene-mask R-CNN to suppress false alarms that appear in the non-target scene area. Zhao et al. [15] proposed an end-to-end aircraft detection frame based on Mask R-CNN to increase the detection performance for the small and dense distributed targets. Su et al. [20] proposed the precise Mask R-CNN based on Mask R-CNN and precise RoI to improve the accuracy of object detection and instance segmentation for very high-resolution (VHR) remote sensing images. Chen et al. [25] proposed a method to calculate the drone building areas based on Mask R-CNN and adopt the concept of transfer learning. Nie et al. [26] proposed a method to achieve inshore ship detection based on Mask R-CNN to ignore the complicated hand-crafted feature. Stiller et al. [27] deployed Mask R-CNN and used a pre-trained model which had been trained with remote sensing imagery to extract large-scale building from VHR airborne RGB images. The above methods are suitable for relatively sparse object in monotonous background. For the various backgrounds and dense object, it is hard to sustain the detection results.

In the present study, we use Mask R-CNN as the framework and propose an improved network model, called SCMask R-CNN, by adding improved self-calibrated convolution (SC-conv) and dilated convolution. We deploy the improved SC-conv to obtain abundant feature which is beneficial to detect and segment dense object under the complex backgrounds. Dilated convolution is helpful to segment the aircraft object in remote sensing images. We test our model on the WFA-1400 dataset. Consequently, the performance is considerably improved, proving that the improved model can be effectively applied to aircraft object detection and segmentation. 

The contributions of our study are as follows: according to the characteristics of aircraft targets in remote sensing images, we add a modified SC-conv to FPN structure to extract richer feature information [7] and a dilated convolution to the mask branch to increase the semantic information of ROIs. The above-mentioned strategies improve the accuracy of Mask R-CNN recognition and segmentation of aircraft targets. In addition, we build WFA-1400 remote sensing aircraft mask dataset as the experiment resource.

## 2. Related Work

We divided the prior work on aircraft detection in remote sensing images into the two main categories of traditional and deep learning methods.

### 2.1. Traditional Methods

Previously, researchers often used methods based on traditional machine learning to solve aircraft object detection in remote sensing images. Traditional machine learning algorithms are roughly divided into four steps: region extraction; feature extraction; feature processing; and classification.

The sliding window method is commonly used in region extraction using sliding windows with different sizes and aspect ratios to slide across the entire image. For the region feature extraction, some specific optimization algorithms have also been proposed. Liu et al. proposed a feature extraction method combining sparse coding and radial gradient transform, in the case of aircraft rotation in the image causing poor detection results [28]. Feature processing principally includes two methods: feature fusion and feature dimension reduction. The methods commonly used in remote sensing image object detection are principal component analysis, Fisher discriminant analysis, and linear discriminant analysis. Finally, the support vector machine, AdaBoost, and conditional random field methods are commonly used in the classification step. However, traditional machine learning methods have the following shortcomings: feature design is highly dependent on professional knowledge; designing suitable, efficient, and robust features is difficult; parameter adjustment is difficult; and the model is relatively solid [28].

### 2.2. Deep Learning Methods

Many remote sensing aircraft detection methods based on neural networks have been proposed because of their strong feature abstraction ability and high accuracy [22,29,30,31,32,33]. Although these algorithms also can be divided into four steps as the traditional methods, all steps are contained in the neural network as an entirety and not divided to many modules. So, the neural network algorithms relieve the laborious hand-crafted feature and data annotations.

In the case of object detection and segmentation, networks can be divided into single- and two-stage models according to the generation stage of the candidate region. The most prominent advantage of the single-stage model is the extremely fast detection speed, while that of the two-stage model is its higher detection accuracy [8,12]. Mask R-CNN can simultaneously perform an end-to-end deep learning model for object detection and instance segmentation. It is an end-to-end deep learning model that means the additional data annotation is needless. In addition, it is equipped with ROI Align instead of ROI Pooling [23]. We select Mask R-CNN as fundamental framework, which is a classical multi-task two-stage neural network.

The author of Mask R-CNN creatively combined Faster R-CNN and FCN [13], which are applied to object detection and semantic segmentation, respectively, by adding a mask branch. The model design structure is pellucid and ingenious. Mask R-CNN can achieve the combination between object detection and segmentation. Instance segmentation refers to the pixel-level classification task. Some pixels contained in the bounding box belong to the background, the rest belonged to the foreground. Semantic segmentation is to judge if a pixel in a scene belongs to a certain class, whereas instance segmentation can be regarded as an extension of semantic segmentation, which further distinguishes each individual object in a scene.

The structure of Mask R-CNN can roughly be described as follows: an image is first passed through the RestNet101 backbone network, and feature maps $\left\{C_{2},C_{3},C_{4},C_{5}\right\}$ with different resolutions are then extracted at different stages to form a “feature map pyramid.” According to the bottom-to-up order, $\left\{C_{2},C_{3},C_{4},C_{5}\right\}$ contains high- to low-level feature information. $\left\{P_{2},P_{3},P_{4},P_{5},P_{6}\right\}$ is obtained through the FPN structure which can get multi-scale feature fusion to increase model’s scale robustness. Based on the generated anchors through RPN, the model performs binary classification (foreground and background) and regression to filter out some proposals. Then, they pass ROI Align to change the ROI into fixed-size 7 × 7 or 14 × 14 px. Finally, the model input ROI into the fully connected layer and FCN for classification, regression, and segmentation.

## 3. Method

**Figure 1:**
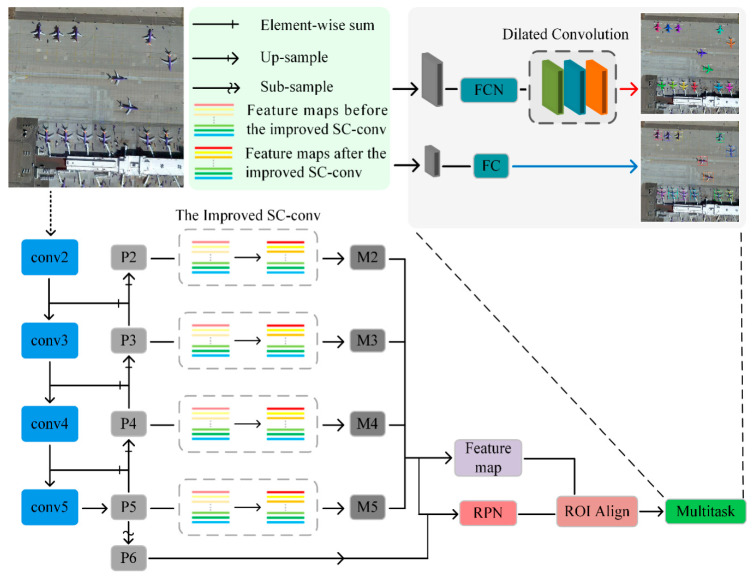
Overall SCMask R-CNN model structure.

**Figure 2:**
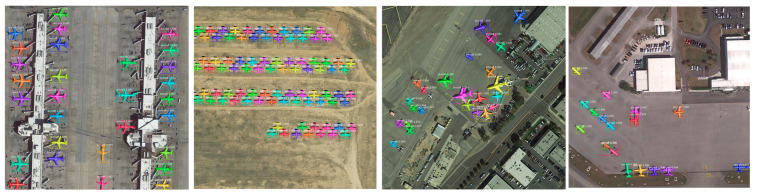
Detection and segmentation performance display in our proposed method.

In this section, we present the overall structure of the network model proposed in Figure 1. The model feeds an input image to the ResNet101 backbone network layer-by-layer to extract the feature maps $\left\{C_{2},C_{3},C_{4},C_{5}\right\}$, through the FPN structure to fuse multi-scale information to obtain $\left\{P_{2},P_{3},P_{4},P_{5}\right\}$, and obtains self-calibrated feature maps $\left\{M_{2},M_{3},M_{4},M_{5}\right\}$ through the SC-conv structure. Finally, the backend of the model performs classification, bounding-box regression, and instance segmentation according to the obtained feature maps. In our model, each bounding box is segmented into aircraft and no aircraft regions (Figure 2).

The features are extracted by the traditional CNN to feed the classification, bounding-box regression, and mask branch at the backend model. It is difficult to extract rich features, due to the small aircraft targets in remote sensing images. This will result in an inaccurate judgment. The deeper the networks grow, the more complex functions the networks can express. Features can be learned from many different levels of abstraction, from the edge (in a lower level) to very complex features (in a deeper level). However, blindly increasing the number of layers of the network will not only impose higher experiment requirements on the hardware conditions, but also the phenomenon of gradient disappearance will become more severe. The training loss will increase instead of reducing, leading to the degradation of the model [34]. Our strategy avoids the method of changing the network structure with a huge resource overhead and turns its attention to the convolution operation. In view of the characteristics of the aircraft targets in remote sensing images, the improved SC-conv is used to self-calibrate the feature information to supplement the missing targets’ edge information in the feature map which is obtained by the ordinary 3 × 3-sized convolution kernel. In addition, we added three dilated convolution layers to the mask branch of Mask R-CNN to further supplement the missing semantic information in each ROI for the instance segmentation.

### 3.1. Improved SC-conv

**Figure 3:**
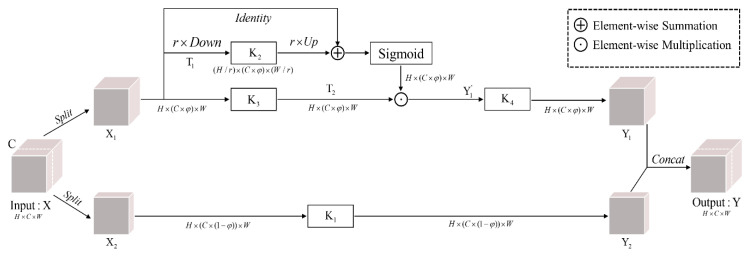
The improved SC-conv structure.

In a normal CNN model, the 3 × 3-sized convolution kernel is commonly used to integrate feature information [34]. SC-conv is different from the convolution with 3 × 3 kernel size [35]. Before convolution, the feature map X with a H×C×W shape must be evenly cropped into two branches according to the number of channels. We refer to them as $X_{1}$ and $X_{2}$, respectively, with the C/2 shape. Figure 3 depicts four sets of convolution kernels in SC-conv denoted as ${\left\{K_{i}\right\}}_{i=1}^{4}$. The SC-conv structure can flexibly control the manner of feature extraction in space by cropping the feature map and setting the convolution kernel size. The $X_{2}$ branch like the 3 × 3-szied convolution kernel with the same resolution as the input is used to extract the original spatial context information, called $Y_{2}$. $Y_{1}$ is obtained after self-calibration through the $X_{1}$ branch. At the end of the SC-conv structure, $Y_{1}$ and $Y_{2}$ are concatenated together to obtain a feature map with more discriminative feature information.

The 3 × 3 kernel size can obtain only limited spatial information due to the limitation of the convolution kernel size, thereby ignoring the information in the larger field of view. This information loss will not have a significant effect in the recognition of natural images, but it appears to be very important in the recognition of small targets in remote sensing images. A small aircraft target may only occupy 32 × 32 px in a remote sensing image, which measures approximately 4000 × 4000 px. Thus, a large loss of learnable features will be observed for networks. We assigned more channels of feature maps to the $X_{1}$ branch to weaken this problem (Figure 3). Increasing the number of channels in $X_{1}$ will extract richer semantic information to supplement the missing target edge information in $Y_{1}$, enhance the target features in $Y_{1}$, and obtain a more discriminative feature map between the foreground and the background. For $\left\{P_{2},P_{3},P_{4},P_{5}\right\}$, the network will gradually lose low-level feature information (e.g., outline and texture) as the number of layers increases, which is critical for semantic segmentation. Therefore, the self-calibration intensity must be increased to accurately locate the aircraft targets without losing basic spatial context information. We denote the channel ratio between $X_{1}$ and X as φ, φ=0.8, corresponding to $\left\{P_{2},P_{3},P_{4},P_{5}\right\}$.

For the $X_{1}$ branch, we first go through an average pooling operation with a size of r×r and a stride of r to obtain the spatial information $T_{1}$ of a larger field of view, as shown in (1):

(1) $$T_{1}=AvgPooling_{r}(X_{1})$$

Second, $T_{1}$ passes through $K_{2}$ convolution and up-sampling in sequence then performs element-wise summation with $X_{1}$ before passing through the sigmoid function. This output performs element-wise multiplication with $T_{2}$ obtained by $K_{3}$ convolution with $X_{1}$, as shown in (2):

(2) $$Y_{1}^{’}=(X_{1}*K_{3})•\sigma (X_{1}+Up(T_{1}*K_{2}))$$

where, ∗ and σ represent convolution and sigmoid function, respectively. Third, $Y_{1}$ is obtained by convolution $K_{4}$, as shown in (3):

(3) $$Y_{1}=Y_{1}^{’}*K_{4}$$

We use the improved SC-conv to extend the 3 × 3 kernel size to two spatial scales: the first is the $X_{2}$ branch with the same resolution as the input X just like a 3 × 3 kernel size; the second is the small size $T_{1}$ after average pooling.

In the $X_{1}$ branch, self-calibration does not pay attention to global information because it inevitably contains information with a negative impact on the current spatial position. On the contrary, the self-calibration focuses on the information around the current spatial position through adjustable scale average pooling. It can obtain a larger field of view to effectively capture the informative context information, fill in the missing low-level feature information, and enhance high-level semantic information for each spatial position. Furthermore, each spatial position can reflect the dependence between channels in a greater extent, as shown in Equation (2).

The improved SC-conv specifically increases the self-calibration convolution intensity in high-level feature maps to strengthen the connection between contexts. In this way, each spatial location contains more informative information and enhances the acquisition of low-level feature information (e.g., clearer texture) in the feature maps.

### 3.2. Dilated Convolution

In the original Mask R-CNN, for the mask branch, the ROIs go through four 3 × 3 convolution layers and then through a transposed convolution in sequence to obtain a 28 × 28 px mask image. Although transposed convolution is better than up-sampling, which is commonly used, it also has a shortcoming of an enlarged image often showing a chessboard effect and losing feature information. Not coincidentally, this deficiency will be magnified in the segmentation for aircraft targets in high-resolution remote sensing images because the loss of information has a huge impact on the segmentation for small aircraft targets. In response to this problem, we added three-layer dilated convolution behind the transposed convolution. Pooling will bring about information loss. In contrast, dilated convolution can obtain a larger receptive field without changing the feature map size, which effectively enriches the feature information. As its name suggests, the convolution kernel has holes, and the dilated rate is an important hyper-parameter that distinguishes dilated convolution from normal convolution operation.

**Figure 4:**
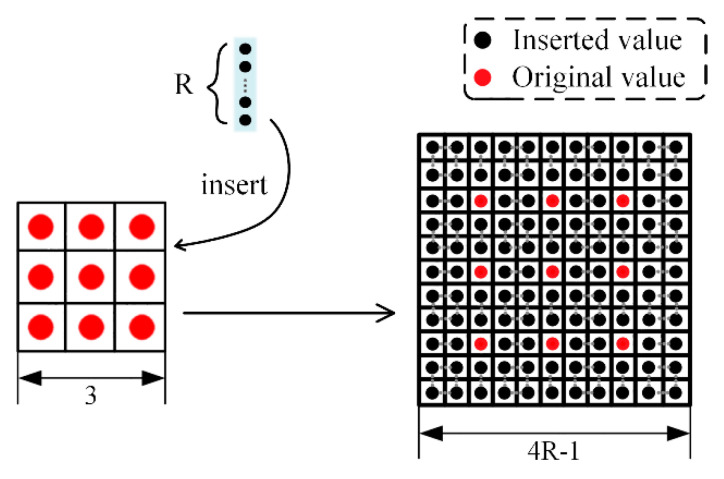
Detailed illustration of the dilated convolution.

The traditional convolution operation 3 × 3 corresponds to a 3 × 3 receptive field, which is not different from dilated convolution with a dilated rate of 1. A dilated rate set to 2 means inserting one zero between two consecutive convolution kernel values along each spatial dimension, which increases the original 3 × 3 receptive field to 7 × 7, making the pixels of the next layer contain a larger field-of-view information. The added three-layer dilated convolution has different hole rates. Figure 4 illustrates the relationship of the receptive field size $S^{’}$, dilated rate R, and kernel size S as $S^{’}=(S+1)\times R-1$. The dilated rate increased as the number of layers increased. We set 1, 2, and 5 to correspond to 3 × 3, 7 × 7, and 19 × 19 receptive fields, respectively, to obtain multi-scale information, reduce the feature loss as much as possible, and achieve a more accurate segmentation for the aircraft targets.

## 4. Experiment

### 4.1. Dataset

Unlike that for natural images, the amount of remote sensing image dataset is relatively small. We created the WFA-1400 remote sensing mask dataset based on the dataset for object detection in aerial images (DOTA) [36] to enable the network to learn more abundant aircraft features. DOTA contains 15 categories (e.g., aircraft, ship, car, and stadium), in which each image is approximately 4000 × 4000 px. Aircraft targets involve a wide variety of types, scenes, scales, and orientations. We selected out all the images containing the aircraft targets in the DOTA dataset and cropped them to 768 × 768 px with an overlap of 64 px (i.e., 1/12) due to the limitation of the GPU memory. We selected the overlap for two reasons. First, it can expand our dataset. Second, it can alleviate the boundary effects at the edges of the input images. The specific implementation in a cropping is as follows: first, we selected 896 × 896 (i.e., 896 = 768 + 64 + 64) px as a big cropping box in an image from the DOTA dataset; second, in this big cropping box, we regarded the central 704 × 704 px as a standard; and finally, we randomly obtained two cropped 768 × 768 px images based on the big cropping box from ±45° orientations. The background generally occupies most of a remote sensing image. If we violently crop images in sequence, we will inevitably obtain many low-quality images (e.g., high background proportion and broken objects). We rotated the cropped image at angles of 90°, 180°, and 270° to expand the dataset capacity. In addition, inspired by Ref. [37], we combined translation, shear, rotation, contrast enhancement, and equalization to further enhance the dataset.

**Figure 5:**
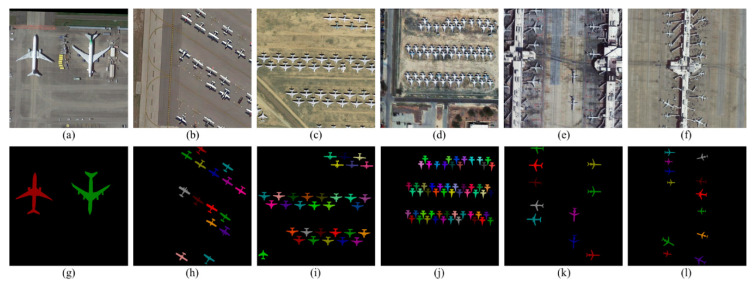
WFA-1400 dataset display. The top row contains six images which are obtained from DOTA. The next column contains corresponding mask image by LabelMe. It colors every aircraft target randomly. (**a**–**f**) Original Image; (**g**–**l**) Mask.

As shown in Figure 5, we used LabelMe 3.16.2 to mark the image with the mask information and generate the corresponding “.json” files. Our dataset contained various types of airliner, warcraft, and glider. Some images had small and dense distributed aircraft targets. Our dataset collects about 7000 aircraft targets (5 targets per image on average) whose size varied from 32 × 32 to 500 × 500 px as show in Table 1. The WFA-1400 dataset collected 1400 images, of which 1120 were used as the training set; 140 were used as the validation set; and 140 were used as the testing set.

### 4.2. Implementation Details

We performed experiments under the Windows 10 operating system using a machine equipped with NVIDIA GeForce GTX-1660Ti GPU (6 GB memory) as the hardware platform. We used Keras as the DL framework for coding and experiments and performed a configuration in the Python 3.6.4 and Keras 2.13.1 compiling environment. Furthermore, we utilized the per-trained ResNet101 model for the initialization. The initial learning rate of the model training was 0.001. The optimization method was stochastic gradient descent (SGD). The momentum was 0.9. The epoch was 50 with 1120 steps per epoch. Hence, the model will go through 56,000 steps. When the epoch reached 20, the learning rate decreased to 10% of the initial learning rate. A smaller learning rate can make the search step of the SGD smaller and avoid the loss function that tends to diverge. Most of the aircraft targets in remote sensing images are relatively small; thus, we allocated five sizes of anchors $\left\{16^{2},32^{2},64^{2},128^{2},256^{2}\right\}$ in the RPN structure. The aspect ratio of the anchor was set to {1:2,1:1,2:1}. We performed all experiments and results under the same training strategy and parameter settings.

### 4.3. Result and Analysis

We used ResNet101 as the backbone network to extract features and the standard metrics to evaluate our results, including AP (average precision), AP_50_, and mIoU (mean intersection over union), and ensure the result validity. They are wide-used and authoritative indicator to judge a deep network model’s performance in object detection and instance segmentation. AP_50_ is the IoU threshold set from 0.50 to 0.95 with a step of 0.05. Table 2 presents the experiment results.

The value of φ is set from 0.5 to 0.9 with a step of 0.1. We called the comparison model with φ=0.8 as Mask R-CNN+05SC. When the value of φ increases between 0.5–0.9, the result gradually rises. Compared with Mask R-CNN+07SC, SCMask R-CNN has a slight improvement. The results of SCMask R-CNN are almost the same as Mask R-CNN+09SC, which show that the result is saturated when φ=0.9.

#### 4.3.1. Mask R-CNN vs. SCMask R-CNN

**Figure 6:**
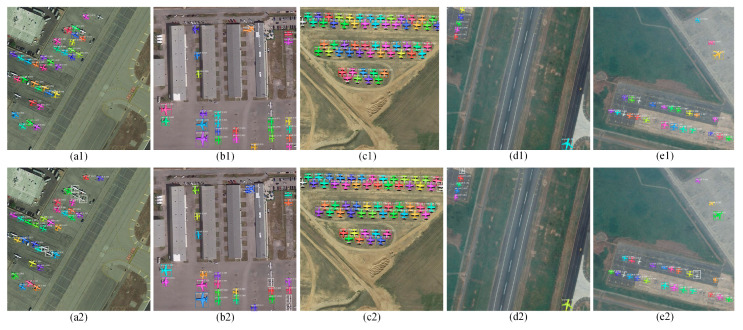
Detection performance comparison. The top row results are based on our method and the bottom row results are based on Mask R-CNN. (**a1**–**e1**) SCMask R-CNN; (**a2**–**e2**) Mask R-CNN

We used the testing set to acquire the model performance. Table 2 presents the test results. The AP, AP_50_, and mIoU of SCMask R-CNN reached 51.7, 96.8, and 72.8%, respectively, which were 1.5, 2.4, and 0.5% higher than Mask R-CNN. The AP_50_ improvement was more significant. In Figure 6, we selected different scenes with different background complexities (e.g., runway, desert, airport, and residential area) and different types of aircrafts (e.g., glider, airliner, and warcraft). The colored boxes and masks represent the results of the aircraft object detection and instance segmentation. In contrast, the white boxes represent the missing detection of Mask R-CNN. In addition, the values of AP on SCMask R-CNN are 84.4%, 88.7%, 96.2%, 98.6%, and 100%, respectively, according to the increasing order of airplane size in Table 1. However, the values of AP on Mask R-CNN are 78.5%, 85.9%, 95.7%, 97.0%, and 97.8%, respectively.

The five sets of images in Figure 6 depict that both methods perform well on medium-sized objects with obvious aircraft structure features (e.g., airfoil shape and engine). In Figure 6c1,c2, Mask R-CNN has a missing detection at the edge of the image. The learnable pixel information is reduced when small objects are at the edge of the image. The weak ability of the feature extraction network will cause missing detection. Therefore, the improved SC-conv structure can obtain a more discriminative feature map by obtaining a larger field-of-view feature information (Figure 6d1–e2).

In Figure 6a1,a2, the detection results of Mask R-CNN show four missing detections compared to SCMask R-CNN. The aircraft targets in the image were gliders, as shown in Figure 6b1,b2. The glider was pulled up by other aircrafts when it took off. The distinction between a glider and other types of aircraft is that it has no engine and has a smaller size. In addition, its airfoil width is relatively narrow (i.e., nearly a rectangle). The airfoils of the other types of aircraft are wide and can be approximated as a trapezoid or a triangle. The glider empennage and airfoil are relatively small, causing features to possibly be lost in high-level semantic information. The small size and the simple structure of the glider will blur its texture features and increase the background interference. Mask R-CNN lost most of the aircraft structural features while extracting the features for these missing detection objects. SCMask R-CNN added an improved SC-conv structure after the FPN to obtain the self-calibration feature map, which enhanced the underlying contour and texture information and the high-level semantic information of the aircraft.

In Figure 6a1,a2, SCMask R-CNN and Mask R-CNN missed an aircraft in the lower left corner. Its shape is similar to other planes. However, the plane is on the corner and above the truck, with complex ground patterns. These details increase the background noise and suppress the Confidence which means that the network does not frame it. The number of planes with such background details is infrequent, making the network sensitive to this background noise.

In Figure 6d1,d2, Mask R-CNN missed an aircraft at the edge of the image. Half of the aircraft was on a light background, while the other half was on a dark background in its bounding box. The texture information was not obvious, causing the spatial and semantic information in the feature pyramid to find it difficult to suppress the background noise. This will consequently result to missing detection. In our method, self-calibration was used for “feature enhancement” (i.e., enhanced the network stability in processing complex backgrounds).

#### 4.3.2. SCMask R-CNN vs. Mask R-CNN+05SC

The SC-conv divided the number of channels of the input X in mean. Mask R-CNN+05SC Table 2 shows that the AP, AP_50_, and mIoU of Mask R-CNN+05SC are 49.9, 95.6, and 71.7%, respectively. Mask R-CNN+05SC showed a better object detection performance over Mask R-CNN. According to [35], AP_S_ dropped from 18.3 to 17.8% when SC-conv was used to segment small objects. SC-conv was harmful to the instance segmentation for small objects. Table 2 illustrates a Mask R-CNN mIoU that is 0.6% higher than that of Mask R-CNN+05SC and 0.5% lower than SCMask R-CNN. The WFA-1400 testing set had some small aircraft targets, leading to a poor instance segmentation performance in the Mask R-CNN+05SC model. A comparison of the results of Mask R-CNN+05SC and SCMask R-CNN showed that the improved SC-conv obtained more discriminative feature maps than SC-conv for the aircraft targets in the remote sensing images. The improved SC-conv had room to fill up the shortcomings of SC-conv in instance segmentation, was slightly better than the Mask R-CNN results, and further improved the object detection performance based on Mask R-CNN+05SC. The improved SC-conv had a stronger feature extraction capability for aircraft targets.

#### 4.3.3. Loss and Training Time

Training our own model took an average of 3800 s and 3–4 s of each epoch and iteration, respectively. When training the original Mask R-CNN model, training the model of each epoch took 3600 s. The time it took for each iteration was almost the same as that of the proposed model. The training time comparison between SCMask R-CNN and Mask R-CNN illustrated that we controlled the increased time of training the model within an acceptable range and obtained better detection results than the original model. Our model reached a trade-off between time consumption and accuracy.

Mask R-CNN is a multi-task model. The loss value is composed of the classification loss, bounding-box loss from the RPN structure, classification loss, bounding-box loss, and mask loss from the backend of the model.

**Figure 7:**
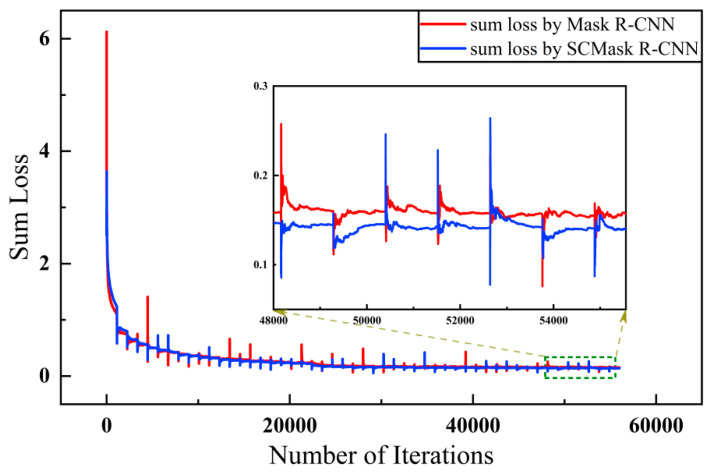
Loss curve. It describes the relationship between the loss and the number of iterations. The training processes of Mask R-CNN and SCMask R-CNN corresponds to the red and the blue curve respectively. We have enlarged the curve in the green box. At the end of training, the blue is lower than the red curve overall, i.e., the loss in our method is smaller.

Figure 7 depicts the loss curves of the two models. The loss value was relatively large in the first training step; thus, the normal graph cannot show the difference between the training conditions of the two models. We zoomed in the part of 48,000–55,500 steps in the graph marked by the green box. The enlarged part illustrates that our model can converge better than the original model. The loss value eventually dropped to 0.141 and 0.154 for our model and the original model, respectively. The proposed model showed a higher convergence level for aircraft targets in remote sensing images.

The experiment showed no satisfactory classification performance for warcraft (e.g., shaped like Chengdu J-20) in the methods (i.e., Mask R-CNN, Mask R-CNN+05SC, and SCMask R-CNN). The reason for which was the lack of remote sensing warcraft samples due to military restrictions. Combining the discussion in Section 3 and Figure 6, the classification performance is strongly related to the aircraft structure. In addition, the aircraft targets, from which Mask R-CNN and SCMask R-CNN detected errors (Figure 6), had some of the following characteristics: the overlap among the aircraft targets was large; the orientation was different; the pixel similarity between the aircraft targets and the background was high; and the aircraft structure features (e.g., aircraft head and empennage) were obscure.

## 5. Conclusions

As an important strategic resource and a mean of transportation, aircraft has a practical value that cannot be ignored in the study of remote sensing images. This study proposed an improved Mask R-CNN model for aircraft detection and segmentation in remote sensing images. We built the WFA-1400 remote sensing aircraft mask dataset and incorporated the modified SC-conv and the dilated convolution into the basic Mask R-CNN model to further enrich high-level feature information and promote the aircraft target detection performance. Our model successfully obtained an improvement of about 2% in the accuracy compared to the basic network. We only paid an acceptable price in time and achieved a significant improvement in aircraft target detection and instance segmentation. Our study has practical significance for the research on remote sensing images. Note that we only performed experiments on the WFA-1400 dataset due to the lack of mature and open remote sensing aircraft mask datasets, which resulted in certain limitations.

## Figures and Tables

**Table 1:** Aircraft size distribution on WFA-1400.

Size/px	0–32	32–64	64–128	128–256	>256
**training set**	1216	2055	1837	530	43
**validation set**	125	132	244	137	10
**testing set**	136	305	135	88	15
**WFA-1400**	1477	2492	2216	755	68

**Table 2:** Detection and segmentation performance of different method.

Method	AP/%	AP_50_/%	mIoU/%	Training Time/h
Mask R-CNN	50.2	94.4	72.3	50.2
Mask R-CNN+05SC	49.9	95.6	71.7	52.5
Mask R-CNN+06SC	50.5	95.9	72.1	52.8
Mask R-CNN+07SC	51.2	96.5	72.7	53
SCMask R-CNN	51.7	96.8	72.8	53.3
Mask R-CNN+09SC	51.4	96.8	72.7	54

## Data Availability

Not applicable.
